# Neuronal processing of complex sounds in the prothoracic ganglion of a bushcricket

**DOI:** 10.1007/s00359-026-01796-3

**Published:** 2026-03-24

**Authors:** Annette Stange-Marten, Maximilian Greif, Steven Abendroth, Manuela Nowotny

**Affiliations:** Animal Physiology Group, Institute of Zoology and Evolutionary Research, Friedrich Schiller University, 07745 Jena, Germany

**Keywords:** Spiking, Local field potentials, Katydids, Insects, Complex sound, Neuronal processing

## Abstract

Male bushcrickets produce complex species-specific songs to attract mating partners. Since the neuronal processing underlying song-pattern detection is not fully understood, we analysed extracellularly recorded song-induced spike responses and local field potentials across the first stage of auditory processing in the prothoracic ganglion of *Mecopoda elongata.* We analysed the influence of the temporal structure and of any change in amplitude of spiking and local field potentials. We presented the complete chirp and compared the neuronal response pattern with those responses evoked individually by each pulse of the chirp. Our data revealed: (i) the complete chirp leads to a transient summation in local field potentials over the time course of the chirp, and (ii) significantly fewer action potentials occur during the chirp presentation than to the individually presented pulses. Our data provide insights into the question of how an acoustical signal can be used to extract those features from sensory information that are important for auditory behaviour, such as chorus formation, species recognition, or phonotaxis.

**Supplementary Information:**

The online version contains supplementary material available at 10.1007/s00359-026-01796-3.

## Introduction

**Fig. 1:**
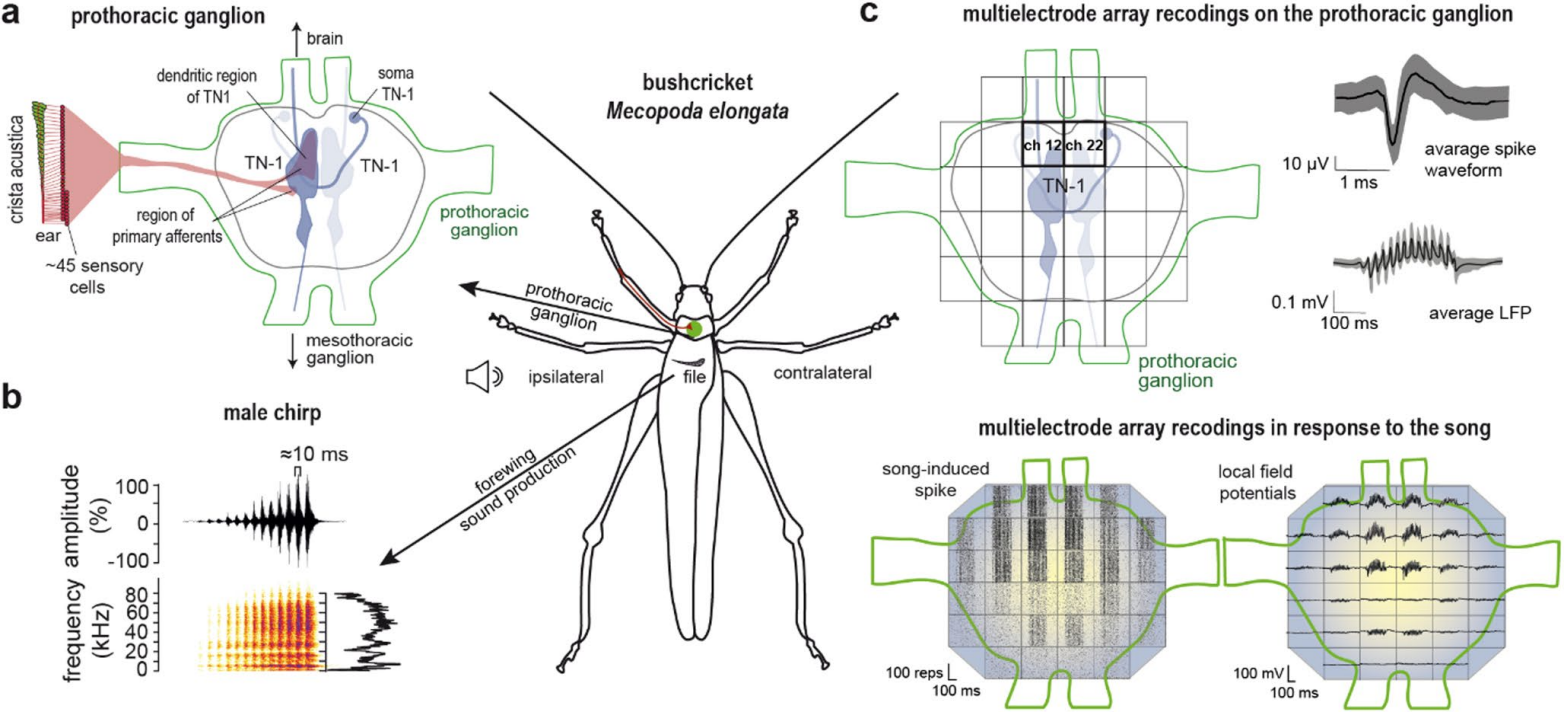
Bushcricket hearing.** a**, The axons of ~ 45 auditory afferents project from the ear in the front legs (crista acustica) to the prothoracic ganglion. The diagram shows the T-shaped bilateral interneurons (TN-1) involved in auditory processing relevant to this study. Please notice that the area where the primary afferents from the ear terminate and the dendritic arborisation of the TN-1 are found overlap in the rostral, medial region of the ganglion. **b**, Sound production by stridulation of the wings in males. The panel displays the normalized amplitude (oscillogram) and frequency spectrum (spectrogram) of a male chirp. Centre panel: Schematic top view of a male *Mecopoda elongata* with labelling of sound-producing and sound-receiving structures and the position of the loudspeaker. **c**, Ventral view of the prothoracic ganglion covered by the 32-channel multielectrode array. The main area with the highest number of spikes and shortest spike latency (Channel 12 and 22) is highlighted. An example of the average spike waveform and local field response to the conspecific song (mean = black line, SD = grey area) is also shown. Below left: Examples (from animal: 190122) of spiking in dot raster plots, and, below right: median local field potentials (*N* = 16) measured in all 32 channels in response to the conspecific chirp; TN = broad-band tuned T-shaped neuron (TN-1)

Animal behaviour can be guided by acoustic signals. Some insects use these acoustic signals to find each other, a process called phonotaxis. For this, the sender encodes messages that the receiver of the signal decodes to identify conspecifics or receptive mates, or even the sender’s position (e.g. Gerhardt and Huber 2002; Schöneich 2020, 2025). Input detection by sensory cells can be important for feature detection at later processing stages. Beside the carrier frequency and amplitude of the signal, temporal patterns also need to be processed during intraspecific social interactions leading to species recognition (Schöneich 2020). In the bushcricket *Mecopoda elongata*, males generate acoustic signals by stridulation of the forewings; females are silent (Fig. 1). The chirps consist of 10–14 broadband pulses (between about 2 and 80 kHz, Fig. 1b) and have a duration about 300 ms (Hummel et al. 2014). Every 2 s, the chirp is stereotypically repeated (Hartbauer et al. 2012a, b; Siegert et al. 2013; Hummel et al. 2017). The pulse repetition rate within each chirp is about 50–60 Hz (Hartbauer et al. 2012a, b). A single pulse in the chirp has a duration of about 10 ms, and pulses increase in level during the chirps (Fig. 1b, Hartbauer et al. 2012a, b; Hummel et al. 2014). Furthermore, *M. elongata* is a chorusing species (Sismondo 1990; Hartbauer et al. 2005), and physical fights, such as those found between male crickets (e.g. Stevenson and Rillich 2012), have not been documented. Their songs merge together in a chorus that attracts conspecific females, which then localize their preferred mate by phonotaxis (Hartbauer et al. 2014, 2015). The structure and level of the song is therefore important for synchronisation between males, and for females to recognise their species and perform phonotaxis.

Bushcrickets have short and tonotopically organized hearing organs (crista acustica) in their forelegs, with a small number of sensory cells, ranging from 15 sensory cells in *Zichya baranovi* (Zhantiev et al. 1995) up to 116 in the male crista of *Ancylecha fenestrata* (Scherberich et al. 2016, 2017). Bipolar afferent neurons from the bushcricket crista acustica carry their sound-induced neuronal responses to the prothoracic ganglion, the first processing centre along the auditory pathway (e.g. Stumpner and Nowotny 2014; Fig. 1a). Previous laservibrometic measurements along the crista acustica revealed that the conspecific song of *M. elongata* induces mechanical movement along the entire hearing organ (Hummel et al. 2014), and this presumably activates all afferent sensory neurons. Therefore, one can speculate that information of the broadband sound of this species lies dominantly in the temporal content, for example in the pulse pattern.

The first thoracic ganglion exhibits a fascinating neuronal arrangement. Neurons that are involved in sending and receiving acoustic signals are activated in this ganglion, in which sound-induced responses are processed by different interneurons (Stumpner and Nowotny 2014; Cillov and Stumpner 2022). In bushcrickets, four types of local interneurons have been described in the prothoracic ganglion. These are the omega, the dorsal unpaired median, local descending, and segmental neurons (Cillov and Stumpner 2022). In *M. elongata*, so far only omega neurons were verified as local interneurons (Römer et al. 2002). Furthermore, there are three types of intersegmental neurons, two of which send their information either to the brain (ascending interneuron, AN), or to the mesothoracic ganglion and beyond (descending interneuron, DN). There are up to five different ANs found in different bushcricket species, and several DN (see for review Stumpner and Nowotny 2014). The third type of intersegmental neurons are the T-shaped neurons (TNs), which transmit information to the brain by an ascending axon, but also down to more posterior ganglia by a descending axon. TNs typically respond with phasic spiking (e.g. Schul et al. 2012) and play an important role in the temporal coding of the conspecific song (e.g. Schul 1997; Siegert et al. 2011).

In *M. elongata*, recordings from the cervical connective were used to monitor axonal activity of the TN-1 (Siegert et al. 2011, 2013). With this technique, it is possible to assess the effects of chirp synchronisation and acoustic masking on the TN-1 spiking, and explain, for example, female choice preference. Recent intracellular studies showed frequency-specific adaptations in the TN-1 of *M. elongata*, and a clear relationship between spiking response and Ca^2+^ signal in the dendritic arborisation of the TN-1 (Bayley and Hedwig 2024). In comparison to pure-tone presentation, the spiking of TN-1 increased during chirp presentation and was logarithmically related to an increase in the mean Ca^2+^ signal. The dendritic arborisation of the TN-1 overlaps strongly with the area where the primary afferents from the ears terminate (Fig. 1a). Beside the broad-band tuned TN-1, there are also other TNs documented in bushcrickets, like the TN-2 in *Tettigonia viridissima*, a neuron that responds mainly to ultrasounds. Since it is fast adapting, TN-2 is assumed not to be involved in intraspecific communication (Schul 1997). Further, Kostarakos and Römer (2015) showed that in *M. elongata* there are at least two other types of TNs in the prothoracic ganglion (i) a T-shaped neuron that responded to broadband frequencies with its strongest responses to higher frequencies (broadband T-1), and (ii) a second type of TN that showed narrow low-frequency tuning (low-frequency T-1). Both of these TNs are activated by the chirp of *M. elongata* and enabled the receiver to a species-specific neuronal response. However, the impact of the single pulses of the chirp on the neuronal responses is not well understood. Therefore, we analysed how the neurons in the prothoracic ganglion responded to the presentation of the entire chirp and to each of the single pulses composing a chirp. Using a multielectrode array placed at the ventral surface of the prothoracic ganglion, we describe here the 2D-spatial distribution of the spiking pattern in this ganglion in response to the chirp and the single pulses. Further, measured as local field potentials (LFPs), the spatial activity of dynamic changes in the electric field around the neurons can be assigned with this technique. The measured LFPs are based on sum potentials of synaptic activity as shown in electroencephalogram recordings of the cerebral cortex activity in vertebrates (e.g. Buzsáki et al., 2004).

## Materials and methods

### Animal and preparation

Since both males and females use the conspecific song for chorus formation (males), species recognition, and, potentially localization (females), we used adult individuals of both sexes of the tropical bushcricket species *Mecopoda elongata* L. (Insecta, Orthoptera, Tettigoniidae; “species S” described by Sismondo 1990; or “Mecopoda sp. 2”, Korsunovskaya 2008). The bushcrickets were kept under constant temperature (24 °C ± 1 °C) and humidity (about 60% ± 5%). All aspects of the research complied with protocols according to the legal requirements in Germany. We recorded neuronal activity in the prothoracic ganglion in 16 (10 female and 6 male) animals. We used another 12 animals (6 female and 6 male) to examine sound-level-dependent spiking. At the beginning of the experiment, the animal was briefly anesthetized with CO_2_ for surgery and the wings, hind- and middle legs were removed. With the ventral side up, the animal was immobilized in a petri dish using Plasticine. A small window was cut into the cuticle/sternite above the prothoracic ganglion to provide access. A flat foil of the multielectrode (FlexMEA 36-OM + µPA32l, Multichannel Systems MCS GmbH, Reutlingen, Germany) that holds 32 recording channels, two reference channels, and two ground-electrode channels (Fig. S1a) was then positioned on the surface of the prothoracic ganglion.

### Experimental design - stimulation and signal recording

We stimulated the bushcrickets acoustically using two different stimulus conditions, (i) either with the entire natural chirp (chirp stimulation), or (ii) with each pulse of the chirp separately. To compare the pulse-induced responses between the chirp and single-pulse stimulation, the individually presented pulses had the same distance to the stimulus onset and level as in the chirp stimulation. The natural sound of the male chirp had been recorded using an Avisoft system (UltraSound-Gate 116Hb basic, Avisoft) with a broadband (20 Hz – 100 kHz) microphone (CO-100 K, Sanken Microphone Co., Ltd, Gruppe 3 GmbH, Germany). The used chirp was a sequence of 13 broadband pulses of increasing levels. The pulse duration and inter-pulse intervals were of about 10 ms each. The stimulus was loaded by a data acquisition board (DAP 5216a board, Microstar Laboratories, Bellevue, WA, USA) and the sound-pressure level adjusted so that the last pulse of the chirp (with the highest level) peaked at 80 dB SPL (PA5, programmable attenuator, TDT PC1, Tucker-Davis Technology, Alachua, FL, USA). Stimuli were amplified (RB-850, Rotel, North Reading, MA, USA) and fed to the loudspeaker (R2904/700000, ScanSpeak, Vidbæk, Denmark) that had been calibrated using a free-field microphone (MK301, Microtech Gefell GmbH, Germany; measuring amplifier type 2610, Brüel and Kjær, Nærum, Denmark). The different stimuli were broadcast with the speaker horizontally directed towards the right acoustic spiracle of the animal (ipsilateral side, Fig. 1) from 20 cm distance. Each stimulus was repeated one hundred times, with a stimulus interval of 2 s between each stimulus. At the end of the experiment, the contralateral frontal leg (i.e. opposite the stimulation side) was severed to remove the sensory input from that side, and the stimulation was repeated.

Due to the small size of the nervous system, classical multielectrode arrays are rarely used in studies of the neurobiology of insects. However, we were able previously to place a flat, 32-channel multielectrode (Fig. S1a) on the prothoracic ganglion and record spiking and LFPs induced by pure tones (Scherberich et al. 2024). The multielectrode recording was triggered by a TTL pulse, and raw signals from the 32 recording channels were collected by a ME32-FAI-µPA System (Multichannel Systems, electrode resistance was about 50 kΩ) with a sampling rate of 25 kHz. All neuronal data were filtered to separate LFPs (< 100 Hz) from extracellularly measured spikes (300–3000 Hz). Evaluation of neuronal responses was carried out using custom-made software in Matlab (v. 7.6.0, The MathWorks, Inc., Natick, MA, USA).

Extracellularly measured spiking typically reflects axonal action potentials of individual interneurons, or compound action potentials in the case of the synchronous spiking of several neurons at the same electrode position. A spike waveform analysis (Multichannel systems, MC-Rack 4.6.2, Multi Channel Systems MCS GmbH) revealed that with the multielectrode array, we could distinguish only one waveform (Fig. 1c). Comparing our spike data with descriptions in the literature (Siegert et al. 2011; Schul et al. 2012) and own data from the neck connective (Fig. S2), we assume that the recorded spiking is mainly generated by the TNs. Arguments in favour of measuring TN spiking include: (i) matching position of the TN soma and arborisation and location where we measured spikes responses in *Mecopoda elongata* (Bayley and Hedwig 2024), (ii) the typically phasic spike pattern (e.g. Schul et al. 2012), (iii) the spike response pattern after removal of the input from the contralateral side with decreasing spike number (Bayley and Hedwig 2024 and iv) spike numbers of about one spike per pulse in response to the chirp (Siegert et al. 2011, 2013; Bayley and Hedwig 2024). Since we found no amplitude differences in the spike amplitude, a separation of TN or AN response using spike amplitudes as possible in neck recordings (Fig. S2a), was not possible with the multielectrode array. In general, hook-electrode recording of the cervical connectives can help to pick up prominent spikes generated by TN-1 (Schul 1997; Faure and Hoy 2000 b, ter Hofstede et al. 2010), perhaps related to a large axon diameter.

### Data and statistical analysis

**Fig. 2:**
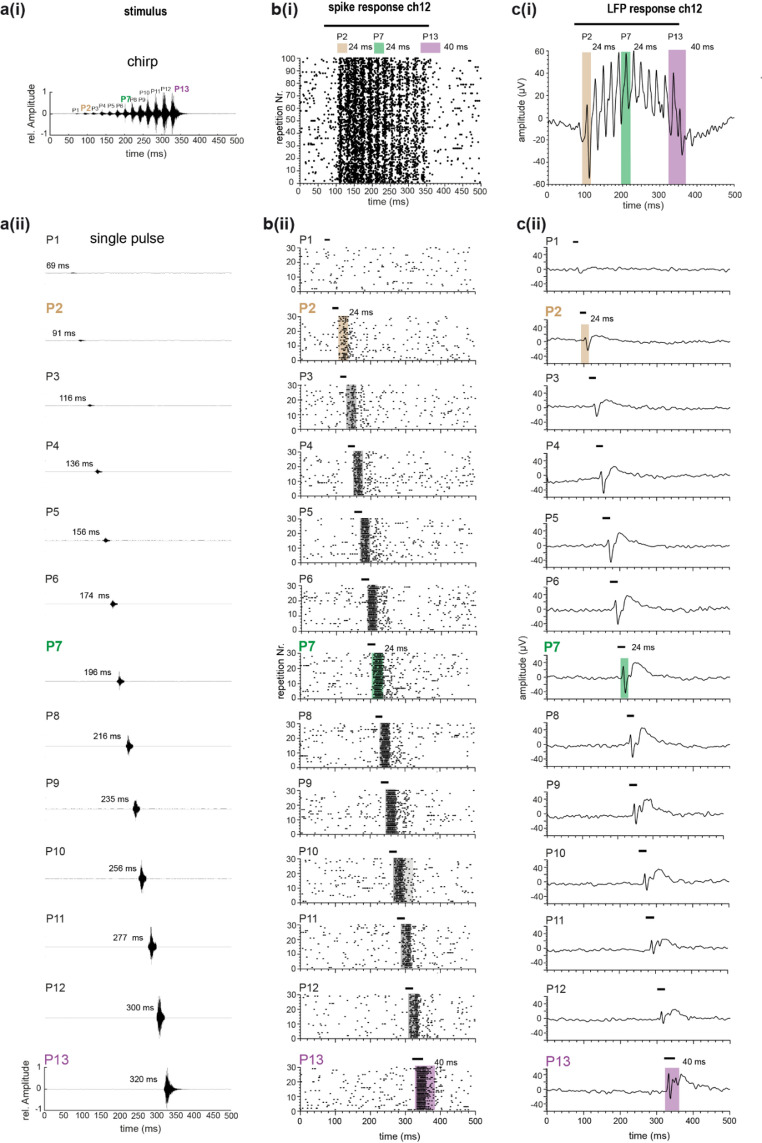
Example of spike and LFP data with time windows of data analysis. **a(i)**, Oscillogram of a chirp and **a(ii)** all single-pulse stimuli (numbers in ms indicate the time point of stimulus onset). **b(i)**, Raster-dot plot of an example of spike responses during chirp and **b(ii)** single-pulse stimulation. The bar above each plot corresponds to the duration of the stimulus. Spike data were mainly analysed for pulse 2 to pulse 12 in a short (24 ms), and for pulse 13 of the chirp, in a long (40 ms) analysis window. **c(i)**, Example of LFP data in response to the chirp and **c(ii)** to single-pulse stimulation. The same duration of the time windows as for the spike data was used. However, the beginning of the analysis was 5 ms earlier. Analysis windows for the comparison of chirp- and single pulse-induced responses are highlighted in brown (pulse 2), green (pulse 7), magenta (pulse 13) and grey (other pulse-induced responses analysed in this study). *LFP* Local field potential, *P* Pulse

For the neuronal response analysis, we measured the spiking and LFP responses in specific time windows of 24 ms for pulse 2 to pulse 12 (Fig. 2). The first pulse of the chirp with the lowest sound pressure did not elicit spiking (Fig. 2b) and was therefore not included in the analysis. Because pulse 13 did not overlap with subsequent pulses, we used a longer analysis window of 40 ms for responses analysis. The start of the time window for spike detection was chosen so that the onset of the stimulus-induced response could be detected. Previous studies indicated that spike responses occur approximately 12 ms after the start onset (Scherberich et al. 2024), which fit well with the measured spike occurrence in the present study. Therefore, the start of each measurement window was set to approximately 10 ms after the start of the pulse (Fig. 2). In Scherberich et al. 2024 it was further shown that spiking activity was delayed by approximately 3 ms to the local field potentials, and is related to a postsynaptic activity. Therefore, for LFP response, the appropriate analysis window started about 5 ms earlier in comparison to the spike response analyses. A threshold was set above the noise level of the filtered responses to define a spike. For the LFP amplitudes, a detection threshold within the averaged response trace elicited by 100 stimulus repetitions was set to RMS+(4xSD) and voltage traces that exceeded this threshold value were accepted as LFPs and used for further analysis.

Data was calculated as the medians across all animals for each recording channel. These medians are presented with interquartile ranges (IQR), or plotted as contour plots (Fig. S1b) using the *contourf* function in Matlab (v. 7.6.0, The MathWorks, Inc., Natick, MA, USA). Recording channels with signals below the detection threshold were not included, and therefore the number of animals used for the calculation of the median values varied. Recording channels with responses below detection threshold are shown as grey areas in the contour plots. Peristimulus-time histograms (PSTH) with a bin width of 2 ms show the occurrence of spikes.

We calculated spike numbers per stimulus as a median value divided by the 100 stimulus repetitions. For spike-rate calculations, we counted the number of spikes generated by the sound stimulation, and then divided the number by the time and number of repetitions to measure the occurrence of the spikes in Hz. The spike latency was calculated by detecting the time difference between the start of the sound pulse and the occurrence of the first spike. For comparison between pulse and chirp stimulation, these starting points were also applied to the chirp-stimulation data. The rate-level gain was determined as RLG = log_10_(spike rate _max_ – spike rate _min_), with the maximal and minimal spike rates being calculated during pulse 13 and pulse 2, respectively.

We calculated beside the maximum and minimum amplitude of the LFPs further the area under the curve from the LFP values. We think that this value of LFP amplitude changes over time (in nVs) better describes the stimulus-induced information flow in a neural network than a simple discrete maximum and minimum values of the LFP amplitudes. For calculating the area under the curve, we integrated the curve between two points (start and ending of the time window). This calculation of the area under the curve was done separately for positive and negative LFP voltages to prevent the positive and negative LFP amplitudes from cancelling each other out. However, the assignment to the original positive and negative amplitudes has been retained in the data presentation.

Statistical analysis was carried out in R (v. 4.0.3) using the ‘dabestr’ package (Data Analysis with Bootstrap Estimation in R, v. 0.3.0). For the comparative analysis, only datasets with at least seven responses (matching the number of individual animals) above detection threshold were taken, and bootstrapping analysis was carried out for each channel separately in R. In the resulting effect plots, the difference between the medians of the different stimulus paradigms, or the different pulses within one stimulus paradigm, defined the effect size. The total effect measured was interpreted as a significant change at an alpha level of 0.05 when the 95% confidence interval did not overlap with zero. To compare the level-dependent chirp with the resulting spike pattern, we generated an envelope of the chirp at different sound pressure levels and the envelope of the related PSTHs. Then we delayed the stimulus by 12 ms, which fits to the delay in the spike response (Scherberich et al. 2024) and led to an overlay of stimulus and response in the time domain. Finally, we calculated the spearman’s correlation coefficient rho (ρ) for stimulus and spiking.

## Results

### Spike coding of the conspecific song in the prothoracic ganglion

**Fig. 3:**
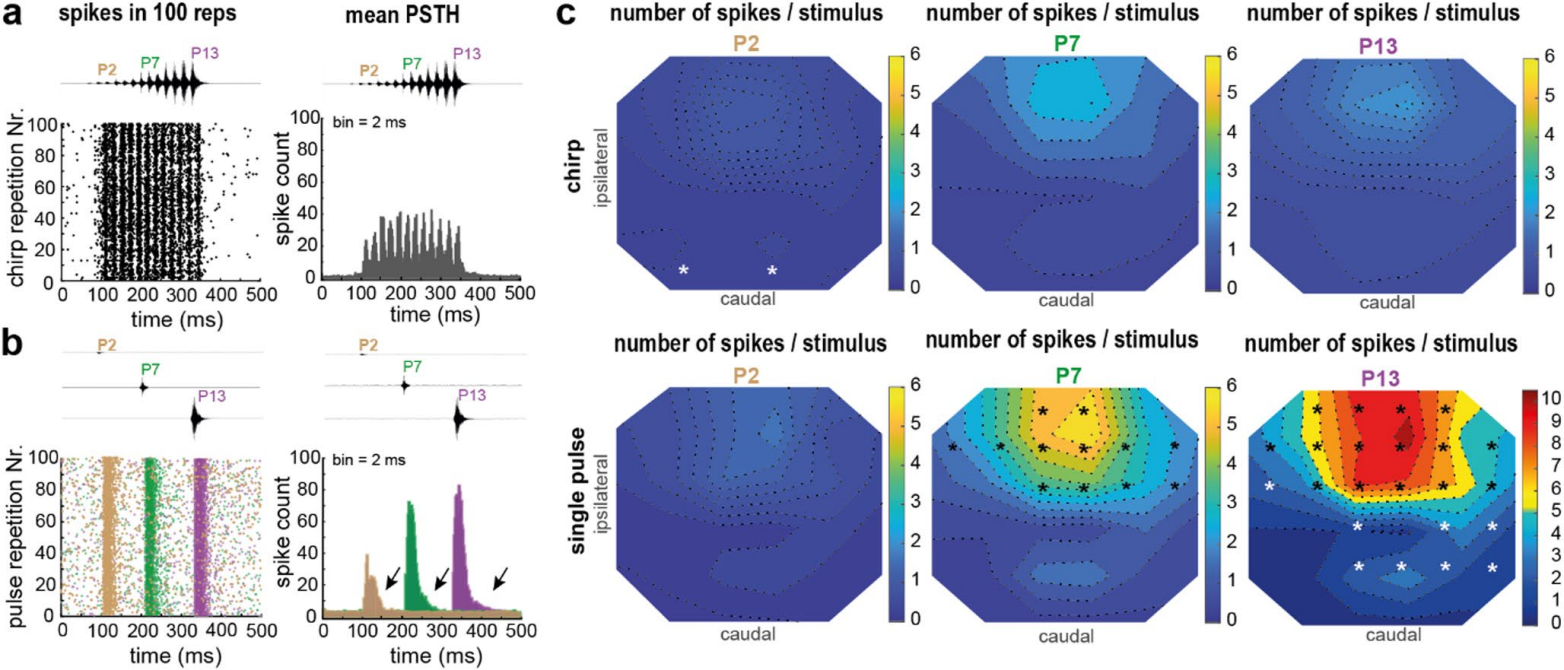
Chirp stimulation evoked a smaller number of spikes in the prothoracic ganglion than single-pulse stimulation. **a**, Raster plot and peristimulus time histograms (PSTH) of an example of spike activity measured at multielectrode electrode channel 12 in response to a chirp stimulus. **b**, Overlaid raster plot and PSTH in response to single-pulse stimulation with pulse 2 (brown), pulse 7 (green), and pulse 13 (magenta). Prolonged spiking activity in response to the single-pulse stimulation is marked by arrows. **c**, Upper panel: Population data of the median number of spikes as a contour plot during the time windows of pulse 2, pulse 7, and pulse 13 within the chirp stimulus (*N* = 16). Lower panel: Population data of the median number of spikes as a contour plot during the presentation of pulse 2, pulse 7, and pulse 13, each as a single pulse. Asterisks indicate electrode channels that had significant differences in the spike number between chirp- and single pulse-stimulation (Bootstrapping Analysis, *p* < 0.05)

We measured the spiking pattern of neurons in the prothoracic ganglion in response to a playback of the male conspecific song of *M. elongata* with a multielectrode array on top of the prothoracic ganglion. The chirp stimulation led to distinct timing in the spike pattern (Fig. 3a, channel 12). The measurement shows an example of spiking at electrode channel 12 (Fig. 3a). The first pulse (pulse 1) with the lowest sound pressure in the chirp did not elicit spiking (Fig. 2b). We compare chirp-induced reposes to early (pulse 2), middle (pulse 7), or late pulse (pulse 13) of the conspecific song (Fig. 3b). When the single pulses of the chirp were presented separately, higher spike numbers per pulse were observed in comparison to the chirp presentation (Fig. 3b), although the stimulus level and time course of the single pulses within a chirp and when presented alone were equal.

Quantifying the 2D distribution of number of spikes in response to each pulse of the song for pulses 2, 7 and 13 either within the chirp or under single-pulse stimulation revealed significant differences in the spike numbers in the rostral, medial part of the prothoracic ganglion during pulse 7 and pulse 13 (Bootstrapping Analysis, *p* < 0.05, asterisks, Fig. 3c). During pulse 2, however, spike numbers were equally low in the rostral medial part (chirp-stimulation, ch12: 1.1 spikes, IQR: 0.5 to 2.1, *N* = 16; single pulse-stimulation, ch12: 1.3 spikes, IQR: 0.7 to 3.4, *N* = 16). The median spike number in response to pulse 7 approximately doubled when the pulse was presented separately (chirp-stimulation, ch12: 2.8 spikes, IQR: 1.6 to 3.3, *N* = 16; single pulse-stimulation, ch12: 5.3 spikes, IQR: 3.0 to 7.4, *N* = 16). For pulse 13 presentation, the spike numbers approximately quadrupled in the single-pulse condition (chirp-stimulation, ch12: 2.0 spikes, IQR: 1.0 to 3.6, *N* = 16; single-pulse stimulation, ch12: 8.8 spikes, IQR: 6.5 to 14.2, *N* = 16). As a control and in comparison to cooling experiments by Bayley and Hedwig (2024), we further measured the number of spikes in a situation of removing the sensory input from the contralateral side (severing the leg at the femur). For pulse 7 and 13, the spike numbers decreased and reached a value of about one spike per pulse during chirp stimulation (Fig. S3). This decrease in spike number was significant for pulse 7 (*p* = 0.038). Please note, during the single pulse stimulation, the spike activity was prolonged compared to the chirp-induced spike activity (Fig. 3b, black arrows). Therefore, when comparing responses between chirp- and single-pulse-induced stimulation, the short time window of 24 ms for pulses pulse 2 to pulse 12 underestimated the spike number for the single-pulse-induced response. The highest activity was on the contralateral side, but there was no significant difference between the activity in different hemiganglia in either stimulation paradigm (ch22 vs. 12, Fig. S1c, d).

### Information coding by spiking pattern

**Fig. 4:**
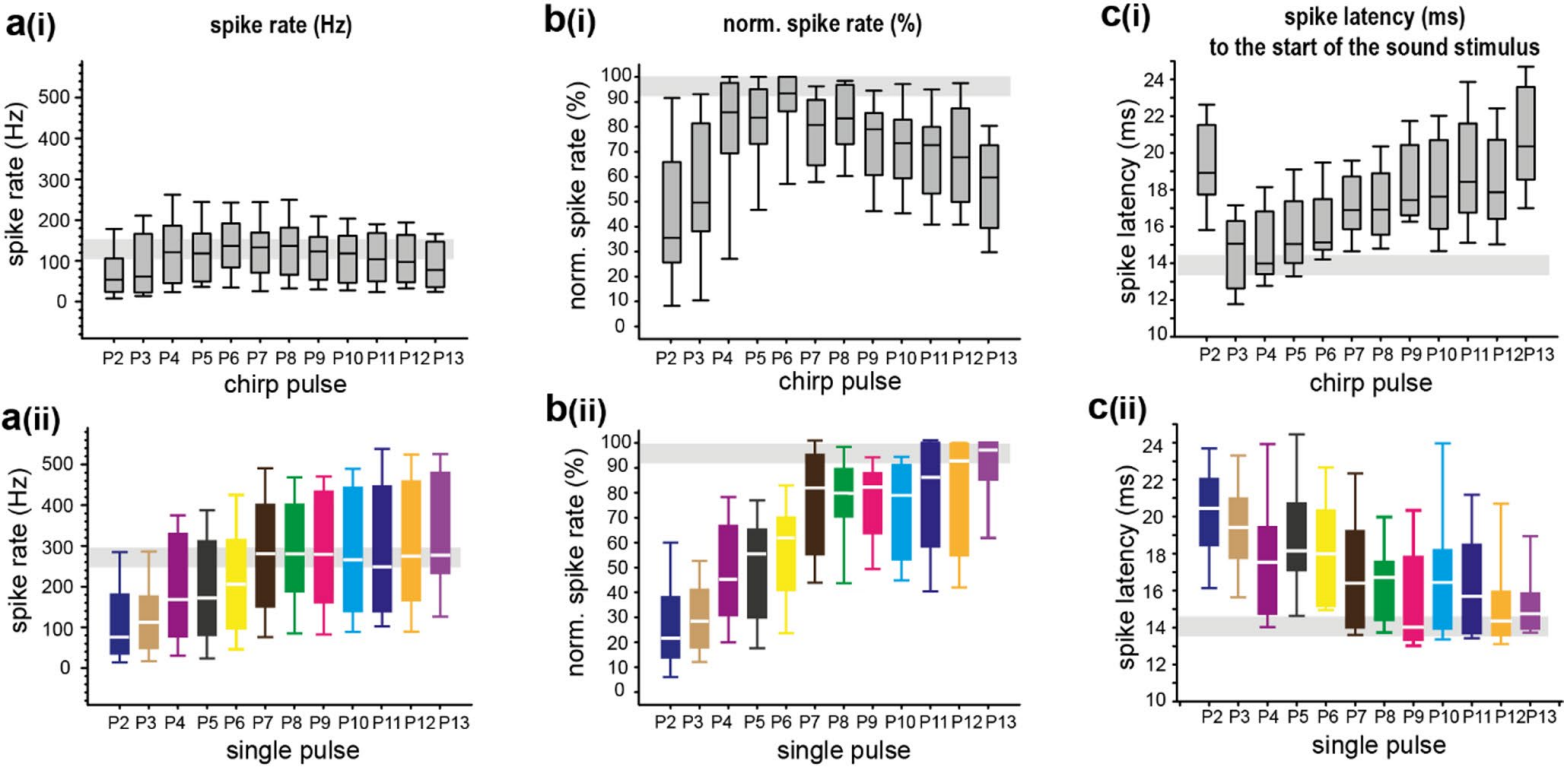
Comparative analysis of spike rate and first-spike latency. **a**, Median spike rates (*N* = 16, electrode channel 12) in response to the conspecific chirp (**ai**) and single pulses of the chirp (**aii**). The grey stripe highlights the range of the maximum median spike rate. **b**, Normalized spike rate for the conspecific chirp (**bi**) and single pulses (**bii**). The grey stripes highlight the maximum spike rate (normalised). **c**, Median spike latency (*N* = 16, electrode channel 12) calculated for each pulse when the entire chirp (**ci**) or the single pulses of the chirp were presented (**cii**). The grey stripes highlight the median of the shortest spike latency

The conspecific chirp has a temporal structure showing multiple pulses of increasing level, constant pulse durations, and intervals of about 10 ms. In response to the natural chirp, we identify a maximal spike rate of 138 Hz within the analysis window of pulse 6 (Fig. 4ai). For the single-pulse presentations, for pulse 6 the median spike rate was 206 Hz (Fig. 4aii). In response to the single pulse 7, the median spike rate was 280 Hz and twice as high as under chirp stimulation (Fig. 4aii). Normalizing the spike-rate data in the natural chirp presentation showed more clearly that the highest spike rates were already reached at pulse 5, and then saturated (Fig. 4bi). The spike rate in the single-pulse presentation increased with the sound level of the pulses, and the maximum was measured for pulse 13 (Fig. 4bii). The gain of the neuronal response across the levels of the pulses was quantified as the rate-level gain (RLG). It was significantly larger during single-pulse presentations than during the chirp (Bootstrapping Analysis, *p* < 0.05, chirp: RLG = 1.89, IQR = 0.83 to 1.94, *N* = 11; single pulse: RLG = 2.33, IQR = 2.12 to 2.47, *N* = 16), showing less-variable spike rates triggered by the chirp.

During the chirp presentation, the spike latency of the first spike in response to the pulses reached a median value of about 14 ms at pulse 4 (Fig. 4ci). At P4 the latencies of the first spike in response to the pulses were longer, and did not decrease further. Specifically, the median spike latency in response to pulses higher than pulse 7 was constant, at 17.5 ms. Under single-pulse stimulation, a spike latency of about 14 ms was measured in response to pulse 12, and the temporal coding was level dependent, with shorter latencies at higher pulse amplitude from pulse 1 to pulse 13. Comparing the spike latency directly between chirp and single-pulse induced responses, the latency was significantly shorter during chirp stimulation in response to pulse 3 up to pulse 6 but strongly significant longer in response to later pulses like pulse 11 up to pulse 13 (Fig. S4).

### Spiking pattern to different sound pressure levels of the chirp

**Fig. 5:**
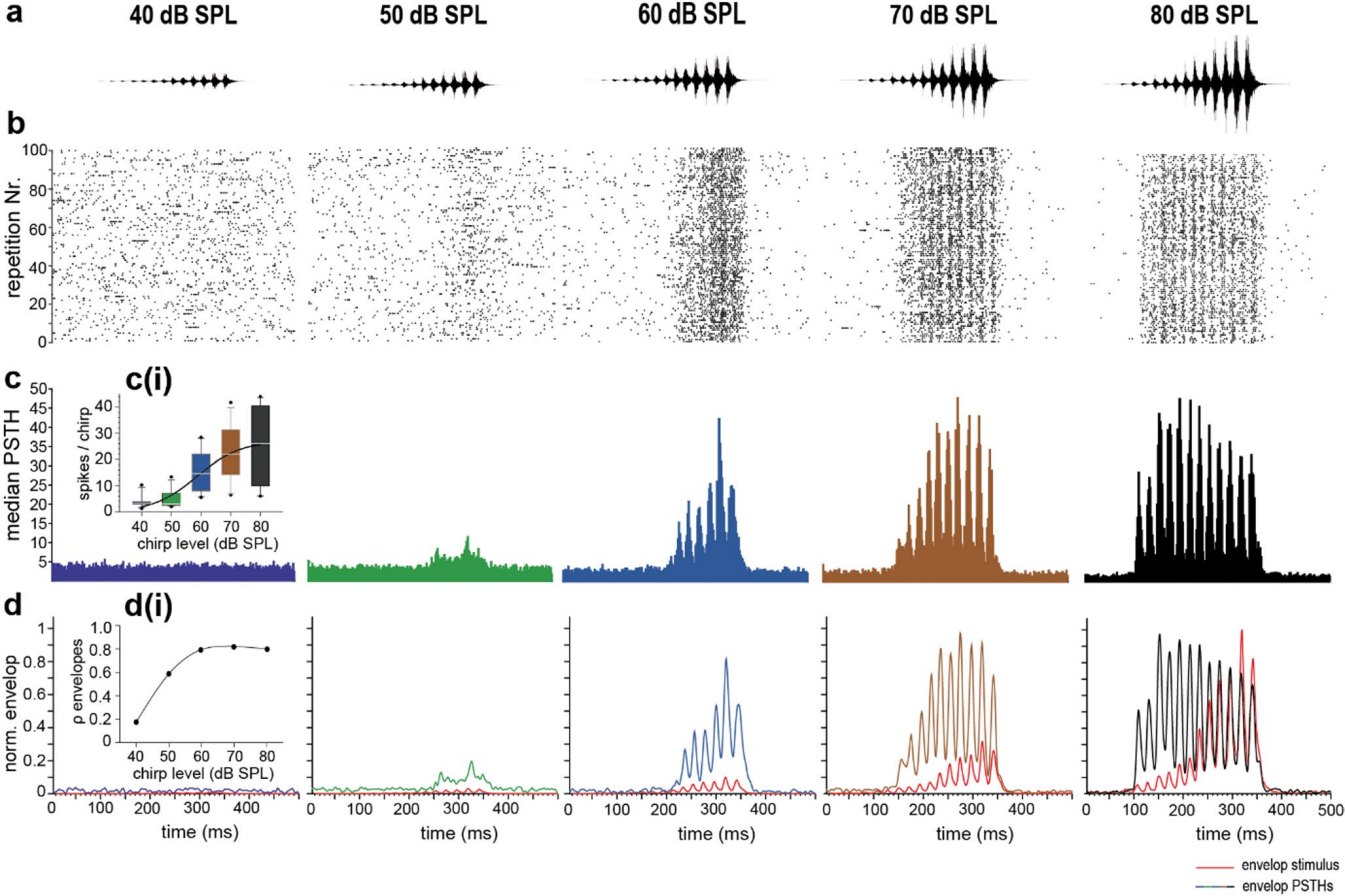
Level-dependent spike responses.** a**, Different levels of the conspecific song (pictograms of the song are only for illustration and not scaled) **b**, Dot raster plots of an example measurement are shown. An increase of the levels of the conspecific chirp led to a more pronounced pattern in spiking. **c**, Median PSTHs of the chirp measured in channel 12 of the multielectrode array (*N* = 12). **c(i)**, The median number of spikes in response to a chirp increase by sound pressure level of the chirp (sigmoidal regression, r^2^ = 0.502). **d**, Median envelope of the chirp and PSTHs. **d(i)** With increasing chirp level there is a stronger correlation (Spearman’s correlation coefficient, ρ = Rho) between both envelopes

Male *M. elongata* need precise temporal information of the song to form a stable leader-follower relationship during the chorus formation, (Hartbauer et al. 2005). Female *M. elongata* use the conspecific song to find the male (e.g. Fertschai et al. 2007; Hartbauer et al. 2014, 2015). During this behaviour, an approach is associated with an increase in the level of the perceived song. By calculating peristimulus time histograms, we show that the temporal structure of the song is preserved with increasing song amplitude, and an increasing number of pulses is represented by the activity of spiking neurons in the prothoracic ganglion (Fig. 5). At a level of 80 dB SPL (at the last pulses), pulses 2 to 13 were individually represented in the spiking pattern and about 26 spikes per chirp are generated (Fig. 5ci). At a level of 70 dB SPL, we found with about 22 spikes per chirp slightly lower spike numbers (Fig. 5ci) and pulses 4 to 13 were individually represented in the spiking pattern. At a level of 60 and lower, the spiking in response to pulses with lower levels vanished, and even less spikes per chirp appear (40 and 50 dB SPL: about 3 spikes/chirp; 60 dB SPL: about 15 spikes/chirp, (Fig. 5c). This increase in the occurrence of spikes and their representation in the temporal pattern of the chirp by increasing sound pressure level was further supported by a correction of the stimulus envelope and spiking (PSTH) envelope (Fig. 5d). The found positive correlation (Fig. 5di) means that as the sound pressure of the chirp increases during a pulse also the spiking in numbers tends to increase (Fig. 5di).

### LFP responses in the prothoracic ganglion

**Fig. 6:**
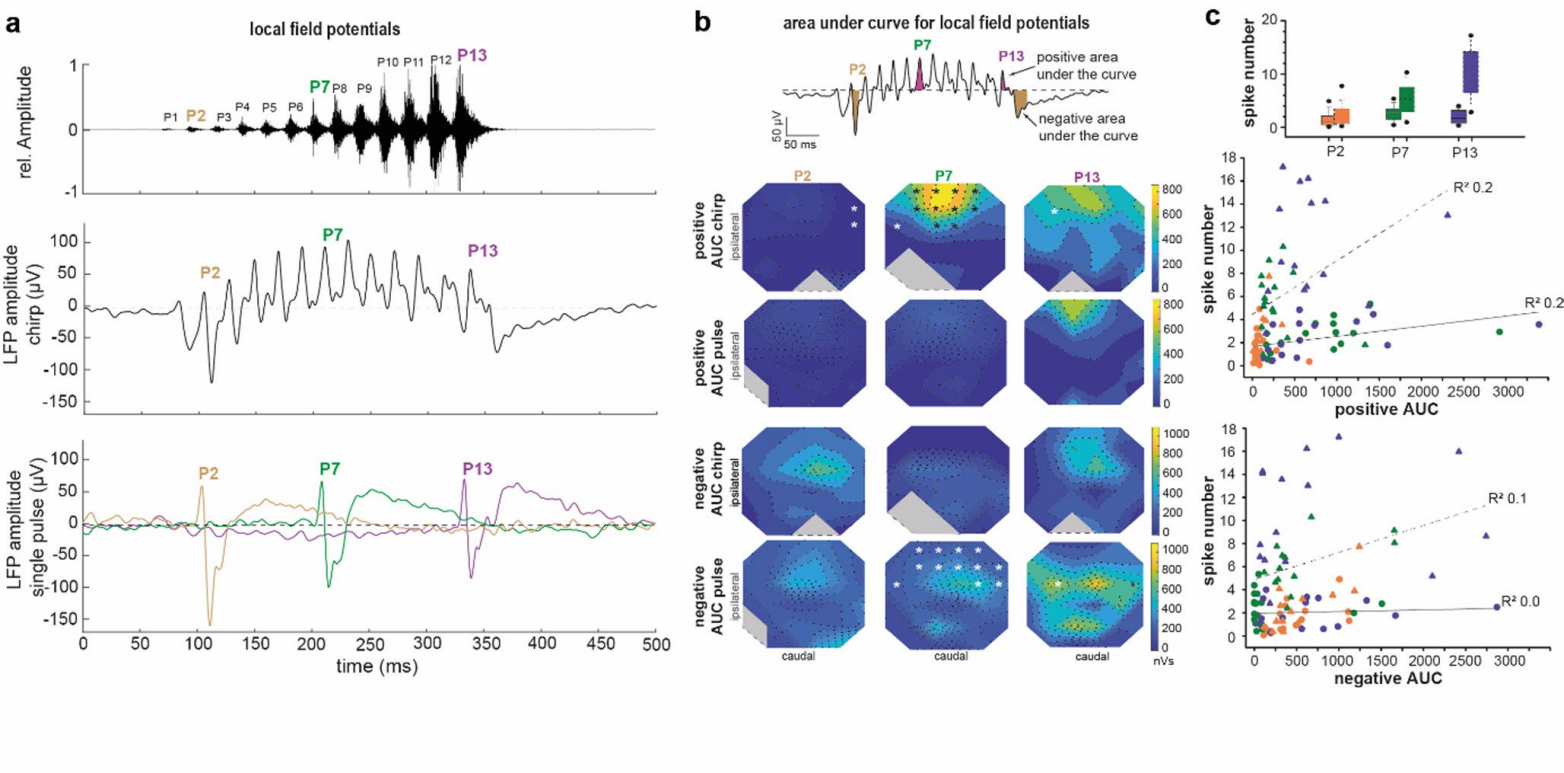
Local field potentials during song processing. **a**, Song-induced LFPs. Oscillogram of the chirp (upper panel). Example median local field potential in channel 12 of the multielectrode array in response to the chirp (middle panel) and single pulses (lower panel). The upper panel displays the chirp stimulus with the pulses marked (1 to 13). Note in comparison to the single pulses the lack of negative amplitudes in the chirp-induced local field-potential response. **b**, Contour plot from the multielectrode array recordings for the area under the curve for positive and negative LFPs in response to the chirp (middle panels) and single pulses (lower panels). The upper panel shows the LFP response to the chirp and the area under the curve for the positive and negative in pink and brown, respectively, for pulses 2, 7, and 13. The analysis time windows are shown in Fig. 2. In the contour plots, signals below the detection threshold (RMS+4xSD) are shown as grey areas. Asterisks point to electrode channels with significant differences in the LFP recordings between chirp- and single pulse-stimulation (Bootstrapping Analysis, *p* < 0.05, N > = 7). **c**, Upper panel: number of spikes in response to pulses in the chirp (solid line box left side) and single pulses (dotted line box right side). Middle and lower panel: Regression between spike number and area under the curve of the LFPs. We analysed the regression between the area under the curve and spike number for pulse 2 (orange), pulse 7 (green) and pulse 13 (purple) for single pulse stimulation (triangles) and chirp stimulation (circles). P = pulse

In both stimulation paradigms, chirp and single pulses of the chirp, every pulse elicited a transient increase in LFP amplitudes. The timing of the single LFP peaks correlated with the timing of the pulses within the stimulus (example shown in Fig. 6a). Induced by the chirp, the overall amplitude of the LFP responses successively increased up to about pulse 8, followed by a decrease in positive LFP amplitudes (Table 1). Interestingly, under chirp stimulation, the LFP amplitude did not decrease to negative values between the pulses in the middle of the chirp. For example, distinct negative potentials were not elicited for pulse 7 (Fig. 6a middle panel). However, we measured a pronounced negative LFP amplitude after the chirp stimulus. When single pulses were presented as separate pulses, each pulse elicited an LFP response with a positive peak (Table 1), followed by a negative peak and a long-lasting positive LFP (Fig. 6a, lower panel). In contrast to the LFP response under chirp stimulation, no reduction of the negative LFP-amplitudes was observed.

**Table 1:** Positive LFP maximum amplitudes measured at channel 12. Amplitude values are given as median (IQR, number of animals). *LFP* Local field potential

	LFP amplitude (µV) chirp	LFP amplitude (µV) single-pulse stimulation
Pulse 2	16.6 (12.6 to 30.9, *N* = 15)	20.2 (10.2 to 29.8, *N* = 14)
Pulse 7	77.0 (39.1 to 101.6, *N* = 16)	46.8 (25.3 to 64.5, *N* = 15)
Pulse 13	64.4 (46.4 to 98.3, *N* = 15)	58.4 (46.4 to 78.9, *N* = 15)

We calculated the area under the curve for pulses 2, 7, and 13 and compared them between the chirp- and single pulse stimulation to analyse the change of the LFP amplitudes over time (Fig. 6b). We found significantly larger areas under the curve for positive LFP amplitudes in the rostral medial part of the prothoracic ganglion under chirp stimulation at pulse 7 than under single pulse stimulation (Table 2). In this region of the ganglion the area under the curve further decreases significantly for the negative LFP amplitudes at pulse 7 (Table 2).

**Table 2:** Calculated area under the curve (AUC) for positive LFP amplitudes and negative LFP amplitudes measured at channel 12. Area values are given as median (IQR, number of animals). *LFP* Local field potential, *P* Pulse

	AUC (nVs) chirp stimulation	AUC (nVs) single pulse stimulation
+ LFP amplitudes during P2	66.3 (44.8 to 127.2, *N* = 15)	87.1 (65.1 to 124.9, N=14)
+ LFP amplitudes during P7	852.5 (221.7 to 1017.7, *N* = 16)	197.0 (128.2 to 338.2, *N* = 15)
+ LFP amplitudes during P13	554.4 (266.7 to 1108.9, *N* = 15)	614.6 (346.5 to 804.3, *N* = 15)
- LFP amplitudes during P2	366.9 (195.8 to 601.3, *N* = 15)	339.2 (263.2 to 570.2, *N* = 14)
- LFP amplitudes during P7	23.9 (0 to 67.6, *N* = 16)	330.3 (253.4 to 461.7, *N* = 15)
- LFP amplitudes during P13	608.1 (153.3 to 946.4, *N* = 15)	325.5 (104.8 to 908.2, *N* = 15)

The LFPs that were used for the area under the curve calculation and the spikes were measured during the same stimulation, and could have had the same neuronal source. We analysed the regression between the positive and negative LFPs for the area under the curve with the number of spikes to test for a possible simple relationship between the area under the curve and the number of spikes. The number of spikes increased significantly from pulse 2 to pulse 13 when all pulses were presented separately, but stayed stable when the pulses were presented in the natural chirp (Figs. 3c and 6c). When we linearly fit the spike numbers to pulse 2, 7, and 13 (individually for each animal) with the positive and negative LFP induced area under the curve, the quantitative effect of positive LFPs on the spike number was low (max. R^2^= 0.2), if not coincidental. Thus, only maximally 20% of the variability of the spike number could be related to the LFPs of the area under the curve (regression analysis for each variable in Fig. 6c). For the regression between the negative LFP induced area under the curve and spike number, the quantitative effect was even lower.

## Discussion

Our data from the bushcricket *M. elongata* revealed neuronal activity that was characterized by reduced spiking responses and reduced negative components in the LFPs during the conspecific song presentation in comparison to the single-pulse stimulation. Our findings further indicate that the neurons monitored were able to code the temporal structure of the chirp. The neuronal representation of the temporal structure of the chirp, could support feature detection in higher brain areas.

What are the sources of the measured responses? We can assume that the neuronal responses depend on the position and size of the segmental and intersegmental neurons. Along the dorsoventral axis of the auditory neuropile, the neurites of ON and TN-1 (Römer et al. 1988) and other auditory neurons (e.g. DUM neurons and ANs) fill the entire space. Therefore, we assume that changes in current flow, measured as LFPs, are picked up from the ventral position of the array electrodes. With the used electrode array, we were further able to measure the spatio-temporal pattern of postsynaptic spiking in response to natural signals. Since previous work in *M. elongata* showed that especially the segmental ON and the intersegmental TN are sensitive to natural broad-band signals, and show a neuronal representation of the chirp signal (e.g. Römer et al. 2002; Siegert et al. 2011, 2013), we focussed here on these two types of bilateral auditory neurons as possible sources. In bushcrickets, ON and TN-1 form dense dendritic arborisations in the anterior region of the prothoracic ganglion (Römer et al. 1988). In the same region, the branches of the primary afferents terminate in the auditory neuropile. Cooling experiments of the ipsi- and contralateral side during intracellular recordings of the ON and TN-1 activity by Bayley and Hedwig (2024) exhibited opposite changes in the spike number of both neurons after the removal of the contralateral input. The spike activity of the ON increased whereas the spiking activity of TN-1 decreased. We also found a strong spike reduction after the removal of the contralateral input, which could be seen as an indication of measuring TN activity. Furthermore, the number of spikes generated in response to each pulse of the chirp decreased to about one/pulse, which confirms the spike numbers of single cell TN-1 measurements in previous studies (Siegert et al. 2011, 2013).

With our multielectrode array, we assume that we recorded the simultaneous spiking responses of bilateral neurons since the channels have an interelectrode distance (centre to centre) of only 300 μm. Even though we report population data here, we can gain information about the processing of acoustic signals by comparing responses between the chirp and single-pulse presentation. Here, we found that under chirp stimulation the spike rate was lower for all pulse-induced responses and the spike latency was shorter for the earlier pulse-induced responses. In contrast, single-pulse stimulation led to a significantly higher spike rate. This spike rate shows a level-dependent increase and shorter spike latencies with increasing pulse numbers from pulse 1 to pulse 13. During chirp stimulation, we found that the responses to the first four pulses were level-dependent in spike rates, and later, with pulse numbers above pulse4, it saturated. This saturation seems to balance the spike number and timing, and so help to represent the temporal pattern of the successive pulses of the chirp, but it eliminates the information on amplitude increase within the chirp. This confirms assumptions that were made in grasshoppers regarding higher levels of the auditory network, where the mean firing rate typically decreases and the response variability increases (e.g. Clemens et al. 2011).

A reduced and less variable spike rate in response to the sound-induced activity of the auditory nerve fibres has also been demonstrated in spherical bushy cells (Keine et al. 2016). These cells are part of the first stage along the central auditory system in mammals. Keine and colleagues described a balance of excitation and inhibition in the spiking of these cells in response to complex sound stimulation (frequency and amplitude modulated and randomized gamma-tone bands) and argued that the rate-level gain control improves the temporal response fidelity mainly by inhibition. On a higher level of the auditory processing like the cortex, inhibitory processes seem to be also important. For example, experiments that compare spiking of cortical neurons in response to natural echolocation sequences and isolated call-echo in bats show forward suppression by the natural echolocation (Beetz et al. 2016). The found suppression is discussed to allow the neurons to more precisely extract the distance information of a target. We also see a smaller rate-level gain during stimulation with the chirp that seems to help to preserve the temporal pattern of the chirp in the spiking.

Beside sound recognition, sound localization is a major task for acoustically orienting animals. In *M. elongata*, sound level could be an important cue to estimate distance to the singer, and thus important for phonotaxis of the female or the positioning of other males during the chorus (Fertschai et al. 2007). The amplitude decay of sound with increasing distance can be detected by the sensitivity of the auditory receptors, as nicely shown in the bushcricket *Mygalopsis marki* (Römer et al. 1987). In the present study we showed that more regular spikes are evoked and more pulses are detectable as the SPL increases. An approaching female that comes closer to the singing male would detect more and more pulses. Therefore, the spike pattern in response to the chirp could act as additional information for phonotaxis. How with increasing levels from pulse 1 to pulse 13 this spike regularity might be used for decoding is, however, still not understood.

Regarding the source of inhibition, the first measurements of thoracic neurons in Tettigoniidae already showed frequency selectivity, directionality, and inhibition (Suga und Katsuki 1961). In the prothoracic ganglion of Tettigoniidae, mechanisms that modify the spike rate were described, like stimulus-specific adaptation (SSA, e.g. Schul 2012). Repetitive signals, such as the pulses in the chirp, were suggested to induce SSA, which could explain the lower spike numbers in the chirp presentation in comparison to the single pulse stimulation. In general, the inhibitory processes during signal processing of acoustic-induced signals, were found in many studies (e.g. Rheinlaender 1975; Stumpner 1999). Furthermore, frequency-specific inhibition is known for many interneurons in several auditory pathways, especially in tettigoniids (e.g. Römer 1987; Stumpner 2002). In orthopterans, different mechanisms were found to describe a reduction in excitability in neurons. For example, presynaptic inhibition could modify spiking activity, as shown in cricket AN2 and lead to divisive gain control (Hildebrandt et al. 2011).

On the postsynaptic side, temporal interaction between EPSP and IPSP in the dendritic branches of the interneurons shape spiking activity (Römer et al. 1988). Furthermore, other intrinsic adaptive functions of auditory neurons were described in crickets (Pollack 1988; Sobel and Tank 1994; Baden and Hedwig 2007) and in bushcrickets (Römer and Krusch 2000; Triblehorn and Schul 2013; Prešern et al. 2015; Bayley and Hedwig 2024). A stimulus-induced Ca^2+^ increase could activate Ca^2+^-sensitive K^+^ channels. This K^+^ shift lowers the membrane potential. Such hyperpolarisation of the membrane potential could lead in ON (e.g. Sobel and Tank 1994; Baden and Hedwig 2007) and in TN-1 (e.g. Triblehorn and Schul 2013; Prešern et al. 2015) to a decreased excitability of the neuron and forward masking of subsequent activation. It is open as to whether such an ion shift could be detected by extracellular measurement techniques, such as the LFPs in multielectrode-array measurements.

LFP responses depend on the electric field around the neurons and are more difficult to interpret than spiking. In a previous study of the neuronal response at the prothoracic ganglion to tonal stimulation, we monitored a long-lasting increase of the LFPs (Scherberich et al. 2024). This net effect in the neuronal activity depended on the sound input over a time of 20 ms, and lasted more than 100 ms after the end of stimulation. In the data presented, we also found long-lasting LFP effects that occur under repeated activation by song pulses of the chirp and show a reduction of the negative LFP amplitudes in comparison to the single pulse presentation. However, since we only found a low correlation between the shape of the LFP response (AUC) and spike numbers, future research needs to focus on the design of new experiments that help to better understand how the field potentials are generated. A detailed investigation of the reduced neuronal response and the sources of excitability of the TN in response to complex signals along the ascending auditory pathway, beginning with the sensory cells itself up to the brain, is another topic for future research.

## Supplementary Information

Below is the link to the electronic supplementary material.

Supplementary Material 1

## Data Availability

Data information is provided within the supplementary information file.
