# Supplementary material for: Neuronal processing of complex sounds in the prothoracic ganglion of a bushcricket
Source: J Comp Physiol A Neuroethol Sens Neural Behav Physiol. 2026 Mar 24;212(3):541–53. doi: 10.1007/s00359-026-01796-3 (PMC13198461; doi:10.1007/s00359-026-01796-3)
Supplement: Supplementary file 1 — Supplementary Material 1 [file 359_2026_1796_MOESM1_ESM.pdf]

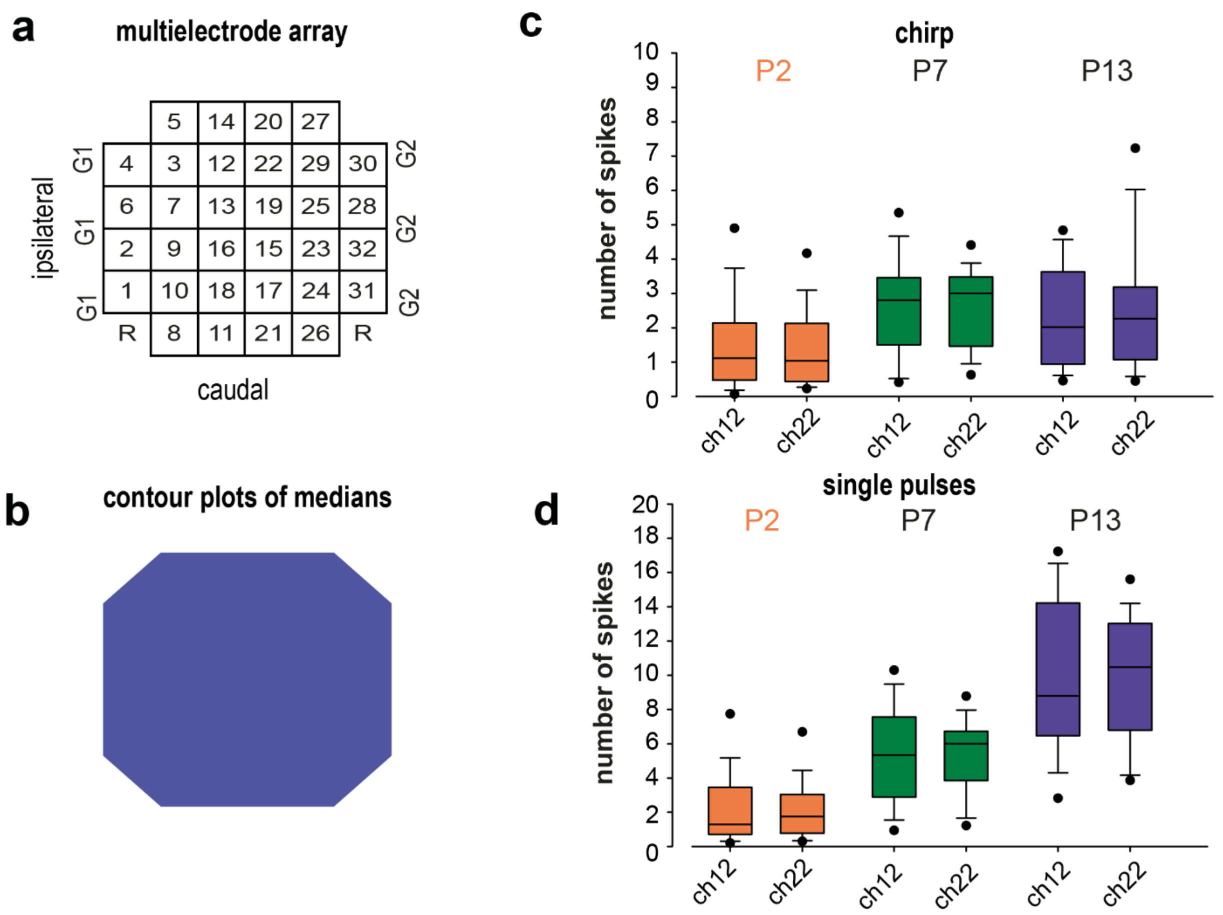

Figure S1. Multielectrode array. a) Flat foil of the multielectrode array with 32 recording channels (1–32), two reference channels (R) and two ground (G). **b**) Medians are plotted as contour plots using the *contourf* function in Matlab (v. 7.6.0, The MathWorks, Inc., Natick, MA, USA). **c**) Comparison of spike numbers in response to chirp stimulation measured in ch 12 and ch22 for pulse 2 (P2), pulse 7 (P7) and pulse 13 (P13) D) Comparison of spike numbers in response to the single pulse stimulation measured in ch 12 and ch22 for pulse 2 (P2), pulse 7 (P7) and pulse 13 (P13). No significant difference ($p > 0.5$; Kruskal-Wallis test) between ch12 and ch22 number of spikes are found.

Chirp ch12 vs ch22

P2: $p = 0.95$

P7: $p = 0.51$

P13: $p = 0.88$

Single pulses ch12 vs ch22

P2: $p = 0.86$

P7: $p = 0.82$

P13: $p = 0.71$

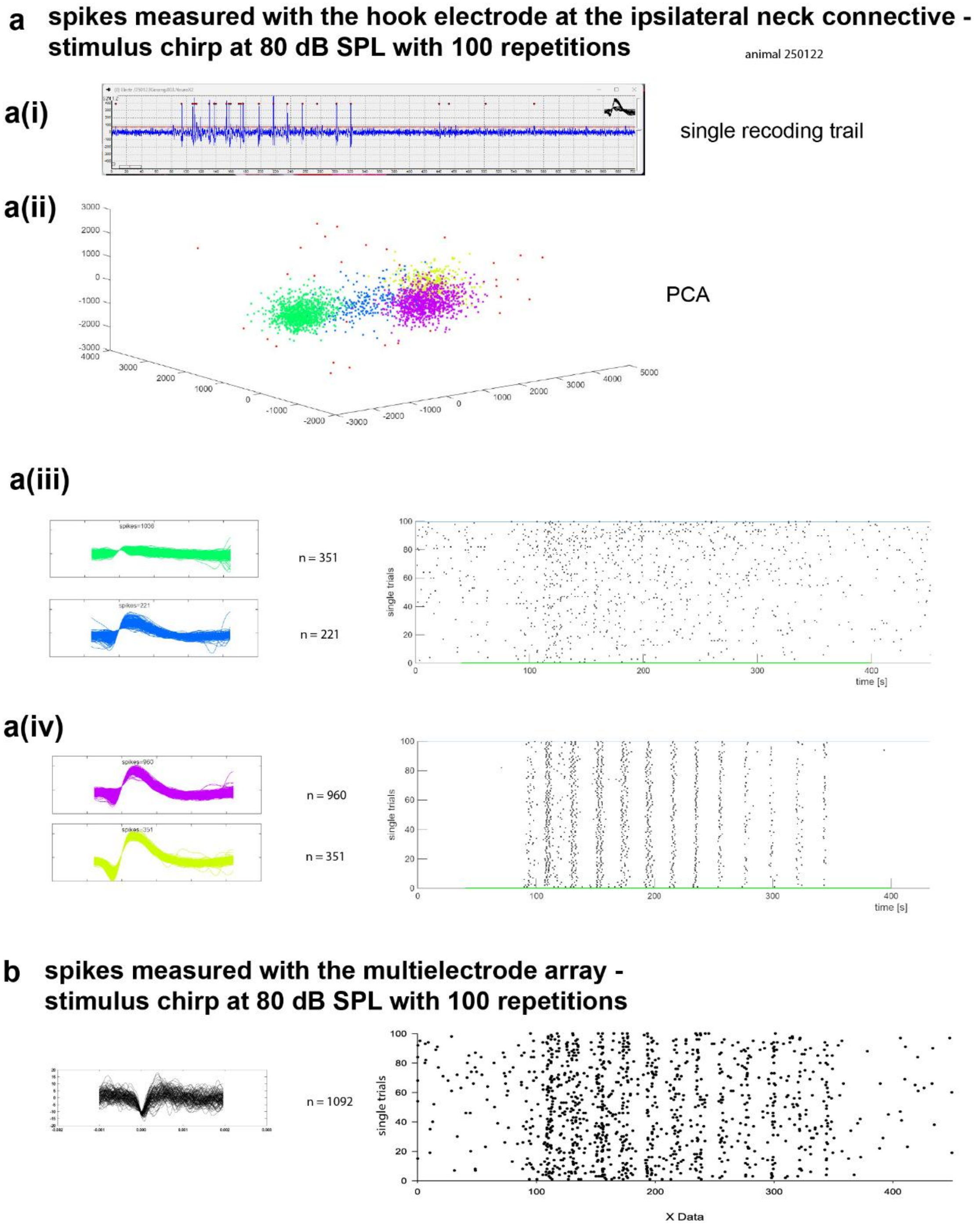

Figure S2. Spike waveform. a) Spike sorting of neuronal data from the ipsilateral neck connective made after the multichannel recording. ai) single trail with spikes in response to the natural chirp. The red points are spikes above the threshold. In the right upper corner, a wave of all spikes is shown. aii) Principle component analysis of all spikes that are measured in response to 100 repetitions of the chirp. The spikes are already grouped for later analysis. aii) Spikes of a closely related waveform (left side) and the resulting dote plot. The tonic non-adapting occurrence points to the activity pattern of an ascending neuron (Schul 1997). aiv) Spikes of a closely related waveform (left side) and the resulting dote plot. The phasic occurrence points to the activity pattern of a T-neuron (Schul 1997). **b**) Waveform of the spikes measured with the multielectrode array at channel 12(left side) and the resulting dote plot. During this measurement the ipsilateral side was only active. The phasic occurrence points to the activity pattern of a T-neuron. *Schul J. 1997. Neuronal basis of phonotactic behaviour in Tettigonia viridissima: Processing of behavioural relevant signals by auditory afferents and thoracic interneurons. J Comp Physiol A 180:573–583.*

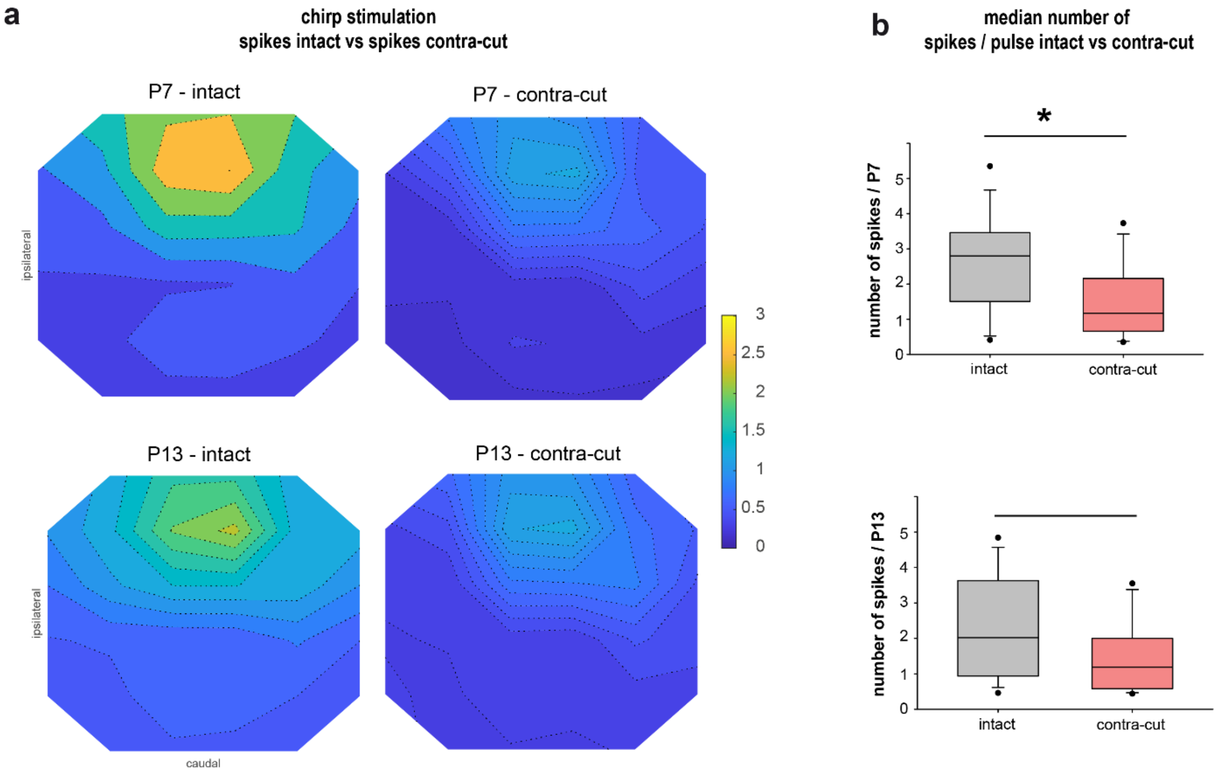

Figure S3. Spike number intact vs contra cut. a) Median total spike numbers in response to pulse 7 (P7, upper panel) and pulse 13 (P13, lower panel). On the left side the spike numbers are shown for an intact preparation and on the left side for a preparation where input from the contralateral stimulation side is cut. **b**) Median spike numbers on a pulse in the intact and contra-cut situation (P7 upper panel, P13 lower panel). An input loss from the contralateral side leads to a decrease of spike number in ch12 (Kruskal-Wallis test; P7 p = 0.038; P13 p = 0.1).

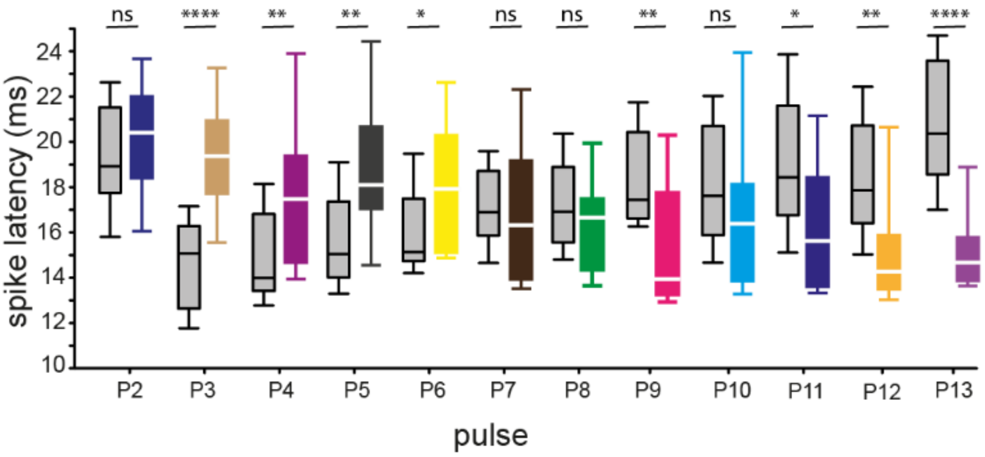

Figure S4. Difference in spike latency between chirp-induced (grey box plots) and single-pulse-induced (colored box plots) response.

Kruskal-Wallis test:

P2, P7, P8, P10: ns

P3: p = 0.000031

P4: p = 0.006

P5: p = 0.002

P6: p = 0.014

P9: p = 0.007

P11: p = 0.016

P12: p = 0.0013

P13: p = 0.000015
